# Singing humpback whales respond to wind noise, but not to vessel noise

**DOI:** 10.1098/rspb.2023.0204

**Published:** 2023-05-10

**Authors:** E. Girola, R. A. Dunlop, M. J. Noad

**Affiliations:** ^1^ Cetacean Ecology Group, University of Queensland, Brisbane, Australia; ^2^ School of Veterinary Science, University of Queensland, Gatton, Australia; ^3^ School of Biological Sciences, University of Queensland, St Lucia, Australia

**Keywords:** communication, anthropogenic noise, Lombard effect, source levels, behaviour

## Abstract

Animal communication systems evolved in the presence of noise generated by natural sources. Many species can increase the source levels of their sounds to maintain effective communication in elevated noise conditions, i.e. they have a Lombard response. Human activities generate additional noise in the environment creating further challenges for these animals. Male humpback whales are known to adjust the source levels of their songs in response to wind noise, which although variable is always present in the ocean. Our study investigated whether this Lombard response increases when singing males are exposed to additional noise generated by motor vessels. Humpback whale singers were recorded off eastern Australia using a fixed hydrophone array. The source levels of the songs produced while the singers were exposed to varying levels of wind noise and vessel noise were measured. Our results show that, even when vessel noise is dominant, singing males still adjust the source levels of their songs to compensate for the underlying wind noise, and do not further increase their source levels to compensate for the additional noise produced by the vessel. Understanding humpback whales' response to noise is important for developing mitigation policies for anthropogenic activities at sea.

## 1. Introduction

Noise is a natural component of most environments, and animals that rely on sound to communicate evolved in the presence of variable levels of noise generated by physical and biological sources. An increase in noise levels can mask animals' signals and reduce their communication range [1]. However, many species can maintain effective communication in high-noise conditions by modifying the acoustic characteristics of their signals (e.g. [2,3]). For example, when the noise increases some animals increase the source levels of their sounds, a phenomenon known as the Lombard effect, to maintain a signal excess above the background noise so that potential receivers can still detect and correctly decode their signals [4].

In the last couple of hundred years, human activities have created additional noise that has substantially changed the soundscape in many regions of the world [5–7]. While some species may be able to use the same strategies developed in natural noise conditions to deal with the additional noise generated by anthropogenic sources, others cannot. In this case, important processes may be disrupted. For example, anthropogenic noise has been shown to interfere with foraging [8,9], mother–offspring interactions [10], anti-predator behaviours [11,12], reproduction [13] and shoaling behaviours [14].

In the ocean, where visual cues can only be used over short distances, many species rely on sounds to fulfil essential tasks, such as coordinating hunting between members of a group [15], capturing prey [16], navigation [17] and courtship [18,19]. While marine animals evolved in the presence of noise generated by natural sources, including earthquakes, wind and waves, precipitations, and other animals such as marine mammals, fish and invertebrates [20–22], they are now also dealing with additional noise generated by anthropogenic activities [5,23]. In most marine environments, shipping is the major source of man-made noise [20,24]. While vessel numbers are variable in space and time, the worldwide increase in shipping activities in recent decades has resulted in overall higher levels of noise in the ocean [25,26] and, in some areas, shipping noise has substantially changed the acoustic characteristics of the environment [6,27,28]. The negative effect of vessel noise on marine species has been widely documented [29–31], and includes stress [32], avoidance behaviours [33,34], changes in diving patterns [35,36] and reduction in foraging success [36]. However, few studies have considered the concurrent effect of natural noise, that although may not be dominant, is always present in the ocean.

In this study, we examine the vocal response of singing humpback whales (*Megaptera novaeangliae*) to vessel noise while accounting for the effect of natural noise. Humpback whales are very vocal animals that produce an extensive repertoire of sounds during foraging [37], social interactions between members of a pod [38], mother–offspring interactions [39] and reproduction [40]. In particular, male humpback whales sing songs consisting of series of stereotyped sounds, called units [40], that have fundamental frequencies between 30 Hz and 5 kHz, and harmonics up to at least 24 kHz [41–44]. This frequency range overlaps with both natural and anthropogenic noise, in particular wind noise [20,22,45] and vessel noise [46–49].

Previous studies have shown that humpback whales are able to modify the source levels of their sounds in response to increasing levels of noise, i.e. they have a Lombard response. For example, Dunlop *et al*. [50] and Dunlop [51] found that humpback whales in eastern Australia increase the source levels of their social sounds in response to increasing levels of wind noise. A similar response has been found for humpback whale songs in both Hawaii [52] and eastern Australia [53]. However, studies on the effect of vessel noise have produced mixed results. While Fournet *et al.* [54] found an increase in the source levels of humpback whale social sounds in Alaska, Dunlop [51] did not find a response to vessel noise in eastern Australia. The effect of vessel noise on humpback whale singing behaviour has not yet been investigated.

In eastern Australia, humpback whales sing on their breeding grounds in the Great Barrier Reef and along their migratory corridor in the coastal waters of New South Wales and Queensland [41,55–58]. The soundscape in these areas is well known and documented [22,50,59,60]. Eastern Australian humpback whales are exposed to variable levels of ambient noise, that is always present in the marine environment. Ambient noise is diffused, homogeneous over large areas and changes slowly with time. While broadband ambient noise includes traffic noise generated by distant shipping, the frequency range of this component falls below 50 Hz and rarely overlaps with humpback whale vocalizations [53]. At higher frequencies the main source of ambient noise is the wind [53]. This shows a broad peak between 100 Hz and 1 kHz that matches the Knudsen curves [21] and overlaps with the frequency range of humpback whale songs [53]. Therefore, in eastern Australia, ambient noise overlapping with the frequency range of humpback whale songs can be considered mainly wind noise.

While historically the Southern Hemisphere has experienced lower levels of shipping traffic compared with the Northern Hemisphere, and levels of vessel noise in waters around Australia are lower compared with other regions [22,25,61], vessel numbers and activity are rapidly increasing in this part of the world [62,63]. Therefore, humpback whales are likely to be exposed to progressively increasing levels of vessel noise in the future [62,64]. Understanding the degree to which singing humpback whales can compensate for vessel noise is important to assess the effect of increasing levels of vessel activities on their communication. Since humpback whale songs are thought to mediate reproductive interactions [65–68], in the absence of coping mechanisms, masking from vessel noise could reduce the reproductive success of the males.

In this study, we compare the Lombard response of male humpback whales to wind and vessel noise. We hypothesize that if the singers increased their source levels only in response to wind noise, we would see the same correlation between source levels of the songs and wind noise levels whether vessel noise is present or not. If the singers had a Lombard response to both wind and vessel noise, we would see a further increase in source levels when vessel noise is added to the environment.

## 2. Methods

### (a) Experimental protocol

Data for this study were collected in September and October 2010 off Peregian Beach, in eastern Australia. At this time of the year, humpback whales travel through the area on their southward migration from breeding grounds in the lagoon of the Great Barrier Reef to feeding grounds in Antarctica. Within our study area, the whales were naturally exposed to variable levels of wind noise that is the dominant component of the soundscape [22,50,59,60]. Additionally, we introduced anthropogenic noise generated by a motor vessel travelling at varying speed and distance. The vessel was a 19 m, 28 ton Westcoaster flybridge cruiser fishing boat, with a fibreglass hull and a diesel inboard engine.

**Figure 1.**
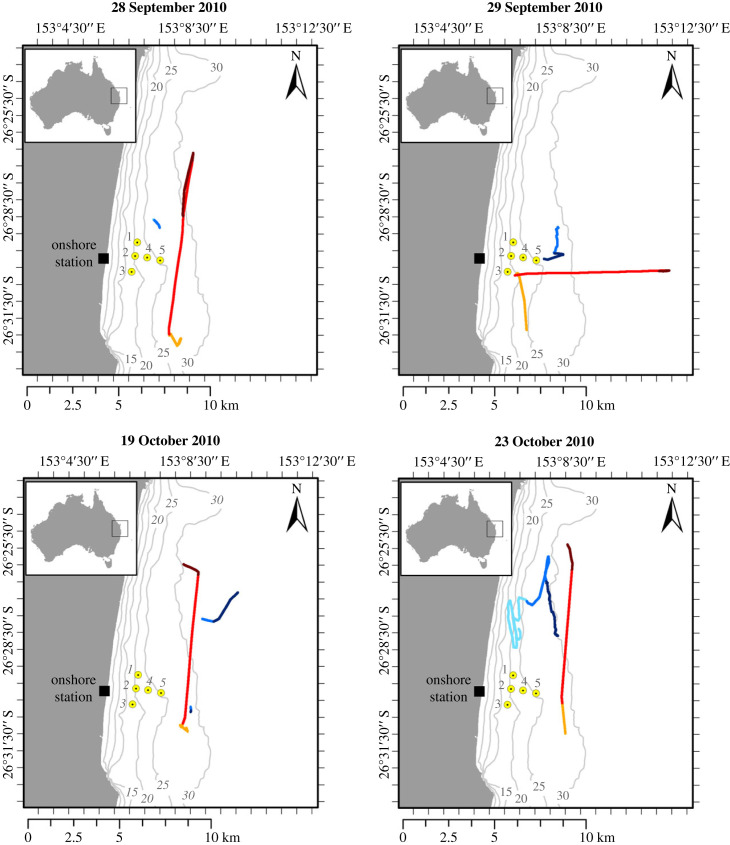
Map of the study site off Peregian Beach in eastern Australia, showing the position of the hydrophone array, and some examples of the tracks of the singing humpback whales and the fishing boat. The hydrophone array is shown as yellow circles and individual hydrophones are identified by numbers: 1–5. The acoustic tracks of the singers are shown in blue and the GPS tracks of the vessel are shown in red. For both vessel (red) and singers (blue), the light shades correspond to the vessel slowly approaching the study area from the south, the medium shades correspond to the vessel travelling along the fixed transects, the darkest shades correspond to the vessel slowly leaving the study area or drifting with the current.

It crossed the study area along two predefined transect lines approximately 8 km long, and oriented northwards and eastwards, i.e. parallel and perpendicular to the general direction of migration (figure 1). The vessel conducted 13 passes throughout the period of the study. During each pass, the vessel approached the area from the south, moving at low speed (less than 1.8 km h^−1^), travelled along one of the two transect lines for 1 h at a speed of 8 km h^−1^, and then moved away from the study area, travelling at low speed (less than 1.8 km h^−1^), or drifting with the current. When the vessel was travelling at low speed and/or was further away, the levels of vessel noise received by the whales usually fell below the wind noise. In this case, the singing males where exposed only to wind noise. However, as the travelling speed of the vessel increased and/or the distance decreased, they were also exposed to additional noise generated by the vessel.

Whale songs were recorded using an acoustic array consisting of five hydrophone buoys arranged in a T-shape configuration and anchored about 750 m from each other. Each acoustic buoy included a HTI-96-MIN hydrophone (High Tech Inc., Long Beach, MS, USA) with built-in +40 dB preamplifier (sensitivity of −164 dB *re* 1 V µPa^−1^ including the preamplifier), a custom-made amplifier, a battery pack and a Magnavox AN/SSQ 41B VHF radio transmitter (Magnavox, Ft Wayne, IN, USA). The signals from the five buoys were received by a Yagi antenna located at an onshore station in Peregian Beach. All the signals were passed through an anti-aliasing filter (−3 dB at 6.5 kHz, −13 dB at 11 kHz and −34 dB at 22 kHz). The signals coming from buoys 1, 2, 3 and 4 were received by a four-channel 8101 Sonobuoy VHF receiver (Australian Defence Force). The signal coming from buoy 5 passed through a custom-built FM down-converter and was received by a Sony FM radio receiver. All signals were then digitized using a PCI-6034E data acquisition card (National Instruments, Austin, TX, USA) and recorded as a multichannel file on a desktop computer, using a sampling rate of 22.05 kHz and a depth of 16 bits.

In our study area, although the soundscape is dominated by the wind [22,50,53,59,60], other noise sources can also occur. These include recreational and fishing vessels, precipitation, invertebrates, fish choruses and background vocalizations from non-focal humpback whales. To exclude the effect of multiple noise sources, recordings were excluded from the analyses: (i) during periods of rain, (ii) for 1 h around dusk when fish choruses are regularly detected, (iii) if more than one whale was vocalizing within 5 km of the array, or (iv) if vessels, other than the research vessel, were observed travelling through the study area by observers located at an elevated location along the coast [53]. These selection criteria ensured that our data included only wind noise and vessel noise generated by our fishing boat.

### (b) Song analysis

Data were processed following the procedures described by Girola *et al*. [69] and summarized here. Analyses were limited to singing whales within 5 km of the array as this provided accurate range estimation and allowed wind noise measured at the hydrophone to be a valid proxy for wind noise at the whale. Individual song units were selected in the channel corresponding to the buoy that was closest to the singer and were saved as individual .wav files. The files were analysed in MATLAB 2016a [70] using a discrete Fourier transform with a Hanning window, 50% overlap and an FFT size of 16 384 samples resulting in a frequency resolution of 1.35 Hz. The background noise was removed using the spectral subtraction technique described by Girola *et al*. [69]. Noise clips for spectral subtraction were extracted from the same channel by selecting the interval between the unit of interest and the previous unit. If after noise removal, the signal-to-noise ratio of the unit at the hydrophone, i.e. the difference between received levels of the unit and received levels of noise, was below 0 dB, the unit was removed from the dataset. For the remaining units, the location of the singer was estimated using the difference in the time-of-arrival of the unit at each buoy. The distance between the singer and the nearest buoy was used to calculate transmission loss using the site-specific empirical model described by Dunlop *et al*. [71]. The unit was trimmed between the times when the energy was between 1% and 99% of the total energy [44]. Root-mean-squared source levels (SL_rms_) were measured on the trimmed signals. To identify the frequency band containing most of the energy of the signal, the 10th centile frequency (F_C10_) and the 90th centile frequency (F_C90_) were also measured. The frequency range between F_C10_ and F_C90_ covers 80% of the energy of the unit. The units were classified into unit types based on the perceived aural characteristics and the distinctive features of their spectrogram.

### (c) Noise measurements

Measurements of wind noise taken before and after each transect were similar, confirming that wind noise levels did not change substantially. Therefore, the wind noise received at the acoustic array before the transect, i.e. when the research vessel was at least 10 km from the hydrophones, was considered a reliable estimate of the wind noise at the location of the singer throughout the transect. To measure wind noise, a 10 s clip was created by selecting parts of the recordings corresponding to intervals without whale sounds between two consecutive song units. The 10 s clips were used to estimate the wind noise for all the song units recorded during that transect. For each song unit within that transect, the noise clip was filtered over the intercentile frequency range of the unit, i.e. the frequency range between its F_C10_ and its F_C90_. Power spectral density level of wind noise (WN_psd_) was calculated on the filtered clip.

The position of the vessel at the time when each unit was produced was recorded using the onboard GPS and was used to calculate the distance between the vessel and the array, and the distance between the vessel and the singer. Vessel noise received at the buoy was measured from the recordings by selecting a noise clip immediately before each song unit. The noise clip was calibrated and corrected for transmission loss between the vessel and the buoy. This provided an estimate of the source level of the vessel. Transmission loss between the vessel and the singer was subtracted from the source level to estimate received level at the location of the singer. The estimated received noise was filtered between the F_C10_ and F_C90_ of the unit. Power spectral density level of the filtered vessel noise (VN_psd_) was calculated. For each unit, VN_psd_ was compared to WN_psd_ to determine which type(s) of noise the singer was exposed to when the unit was produced: wind and vessel noise, or wind noise only. For the units produced in the presence of both wind and vessel noise, the difference between VN_psd_ and WN_psd_ was calculated. This represents the vessel noise excess (VNEX) above the underlying wind noise. Only units produced when VNEX > 3 dB were considered for the analysis of the effects of vessel noise, i.e. the received levels of vessel noise at the singer had to be at least 3 dB higher than the wind noise. The 3 dB threshold corresponds to double the sound intensity and ensured that the difference between the two noise types was well above the error of measurement for our system.

### (d) Statistical analyses

When testing for a Lombard response, as noise levels increase a higher proportion of low-level sounds may be excluded from the analyses due to masking [50,72]. Therefore, signal source levels can artificially appear to increase as the noise increases. To check this, both the dataset containing units produced while the singers were exposed only to wind noise and the dataset containing units produced when the singers were exposed to both wind and vessel noise were split into three groups covering equal ranges of received noise (WN_psd_, VN_psd_): low, medium and high. For each group, the source level of the units (SL_rms_) was tested for skewness using a D'Agostino test [73]. A right skew in the high-noise groups would suggest that the subset is missing a high proportion of low-level sounds and is biased towards higher SL_rms_.

Changes in the source levels of song units in response to variable levels of noise were tested using generalized additive mixed models using the *mgcv* package in R [74]. Separate models were created to analyse the two datasets and then compared. Each model included the source level of song units (*SL_rms_*) as the response variable; while *unit type*, the distance between singer and hydrophone (*r*), singer's ID (*singer*) and noise levels were the explanatory variables. *Unit type* was included to account for stereotypical differences in source levels [41,44,69,75,76]. Only unit types with more than 15 samples were included in the analysis. The distance, *r*, was included as a nonlinear component to account for loss of high-level sounds from singers located closer to the array and whose received level exceeded the functional limit of the recording system and loss of low-level sounds produced by singers located further from the array [69]. Since multiple units were measured for each whale, *singer* was included as a random effect to account for inter-individual variability. Since wind noise is always present in the marine environment, and the source levels of the units have been shown to be positively correlated to it [53], *WN_psd_* was included in all models. Girola *et al*. [53] have shown that for levels of wind noise typical of our study area, the increase in the source levels of the song is linear and does not reach the upper limit of the sound production system. Therefore, *WN_psd_* was modelled as linear. For the dataset containing units produced when the singer was exposed to both wind and vessel noise, the vessel noise excess, *VNEX*, was included in the models to test whether the singers showed an additional Lombard response. The variables were modelled using a Gaussian distribution and an identity function. Following the procedures described by Zuur *et al*. [77], *p*-values were used to assess the significance of the variables. Akaike's information criteria (AIC) were used to select the most parsimonious model between the full model and a series of sub-models where the variables were removed one at a time. To assess model fit, plots of residuals were inspected and residuals were tested for normality and overdispersion.

It should be noted that previous studies investigating humpback whale response to vessel noise [51,54] did not differentiate between the dominant vessel noise and the underlying wind noise. Instead, when vessel noise was dominant, they assumed that it was the only noise the whales would respond to, and did not account for underlying variable levels of wind noise. In our study, by using the vessel noise excess we could model at the same time variable levels of underlying wind noise and changing levels of vessel noise. We also considered that vessel noise can be produced at higher levels compared to wind noise [25], and singing humpback whales may only be able to compensate for it up to a certain point. Therefore, *VNEX* was modelled as producing both a linear response and, to investigate whether an upper limit in the Lombard response was reached, a nonlinear response.

## 3. Results

### (a) Source levels of humpback whale songs and noise characteristics

**Figure 2.**
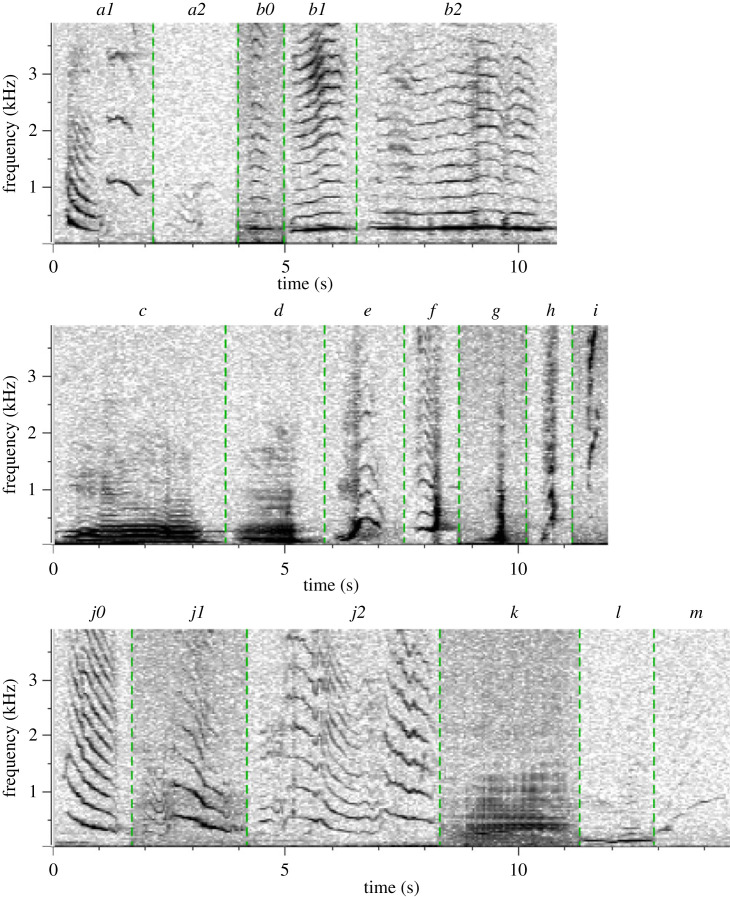
Spectrogram of the unit types identified in the 2010 humpback whale song recorded off Peregian Beach, eastern Australia, during the whales' southward migration. A total of 18 different unit types were identified. A sample for each unit type is included. Samples were extracted from the original recordings and pasted together to generate this figure, i.e. the figure does not represent the order in which the different unit types appear in the song.

A total of 1441 song units were extracted from the recordings and measured. Their SL_rms_ varied between 150 and 189 dB *re* 1 µPa at 1 m (mean 173; s.d. 7.3). The units were classified into 18 unit types (figure 2). Mean SL_rms_ varied by up to 21 dB between unit types.

**Figure 3.**
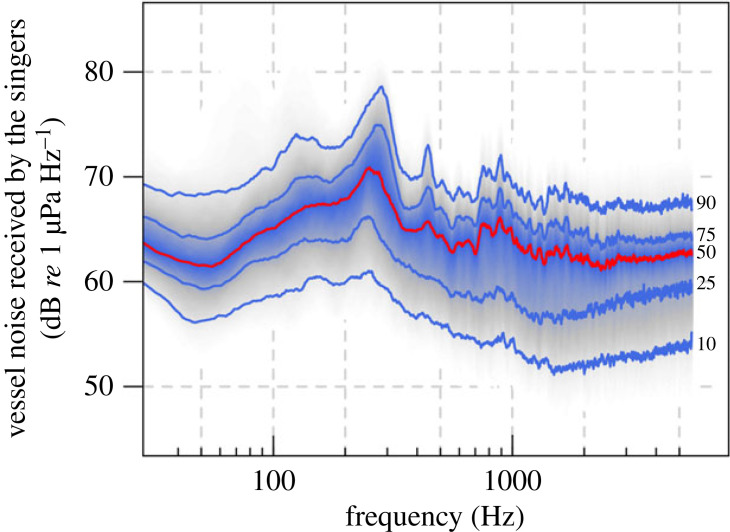
Power spectral density of broadband vessel noise estimated at the location of the singers. Recordings were taken off Peregian Beach, eastern Australia, during the 2010 southward migration. Received levels of vessel noise at the singers were estimated immediately before each song unit was produced. Estimates were obtained by: (i) measuring vessel noise received at the hydrophone; (ii) adding transmission loss between the hydrophone and the vessel to calculate the source levels of the vessel; (iii) subtracting transmission loss between the vessel and the location of the singer. The plot was created by calculating the statistical distribution of received noise levels for each frequency bin. The red line, marked as 50, corresponds to the median (50th percentile). The blue lines, marked as 10, 25, 75 and 90, show the 10th, 25th, 75th and 90th percentile, respectively.

Of the 1441 selected song units, 429 were produced when the singers were exposed only to wind noise, and 501 were produced when both wind and vessel noise were present and received levels of vessel noise were at least 3 dB higher than wind noise levels. For units produced when the singers were exposed only to wind noise, WN_psd_ varied between 50 and 70 dB *re* 1 µPa Hz^−1^ (psd), with a mean of 61 dB *re* 1 µPa Hz^−1^ (s.d. 3.2). While for the units produced in the presence of both wind and vessel noise, the underlaying wind noise (WN_psd_) varied between 50 and 70 dB *re* 1 µPa Hz^−1^ (psd), vessel noise (VN_psd_) estimated at the location of the singer ranged between 54 and 78 dB *re* 1 µPa Hz^−1^ (psd), and the noise excess (VNEX), i.e. the difference between vessel and wind noise, was between 3 and 22 dB. Power spectral density of broadband vessel noise estimated at the location of the singers is reported in figure 3. For a comparison with a typical power spectral density plot of broadband wind noise for our study area, see Girola *et al*. [53].

### (b) Lombard effect

The dataset containing units produced when the singers were exposed to both wind and vessel noise was split into low- (from 53.9 to 62.1 dB *re* 1 µPa Hz^−1^), medium- (from 62.2 to 70.3 dB *re* 1 µPa Hz^−1^) and high-noise groups (from 70.4 to 78.5 dB *re* 1 µPa Hz^−1^). The source levels of the units (SL_rms_) did not show any skewness in any of the groups (D'Agostino test = 0.22 for the low-noise group, D'Agostino test = 0.11 for the medium-noise group, and D'Agostino test = 0.26 for the high-noise group). The dataset containing units produced while the singers were exposed only to wind noise was also split into low- (from 50.4 to 56.9 dB *re* 1 µPa Hz^−1^), medium- (from 57.0 to 63.4 dB *re* 1 µPa Hz^−1^) and high-noise groups (from 63.5 to 69.9 dB *re* 1 µPa Hz^−1^). There was no skewness within these groups either (D'Agostino test = −0.67 for the low-, 0.18 for the medium- and −0.39 for the high-noise group). This suggests that even in high-noise conditions the datasets were not biased towards high source level sounds.

**Figure 4.**
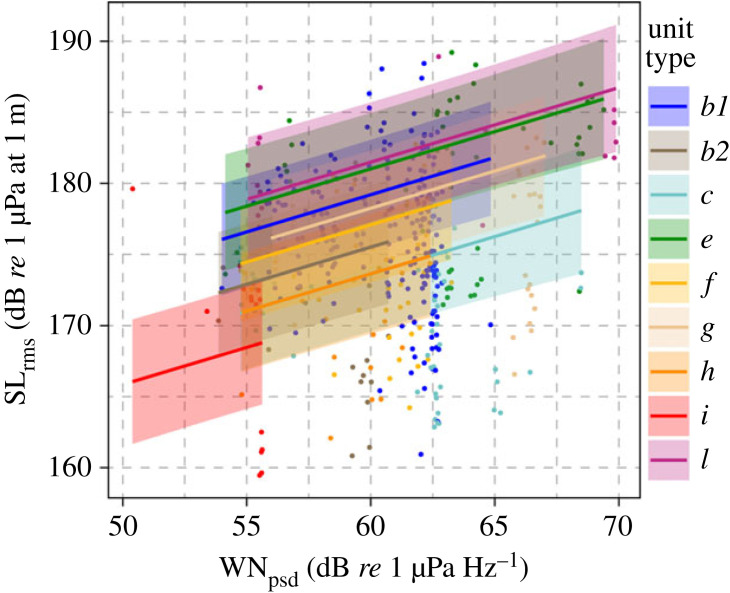
Plot showing the relationship between source levels and noise levels for units produced when only wind noise is present. Each unit type is represented by a different colour, with mean predicted values plotted as solid lines. The shaded areas represent the 95% pointwise confidence intervals. Observed values are shown as dots. The effect of the distance from the hydrophone, *r*, is not shown which leads to the observed data appearing spread in this plot. The plot shows that source levels of humpback whale songs (*SL_rms_*) are positively correlated to wind noise (*WN_psd_*).

For the dataset containing only wind noise, nine unit types were present with a large enough sample size (*N* ≥ 15) to model the effect of increasing noise levels on the source level of the song. Results of the statistical analyses for this dataset show that wind noise (*WN_psd_*) was statistically significant (*p*-value = 1.79 × 10^−8^), and contributed to the model (AIC for the model including *WN_psd_* = 2552.252; AIC for the model without *WN_psd_* = 2577.98). Therefore, the best model for this dataset included *unit type*, *singer*, the distance between the singer and the buoy (*r*), and *WN_psd_*. For this model, plots of residuals did not show any distinctive patterns. No overdispersion was found. Results show that humpback whale singers increased the source levels of their song units by 0.52 dB for an increase of 1 dB in wind noise (table 1 and figure 4).

**Table 1.** Parameter estimates and *p*-values for the model testing for a correlation between source level of humpback whale songs and noise levels for song units produced when only wind noise is present. In this model, source level of the units (*SL_rms_*) is the response variable; while unit type (*unit type*), singer ID (*singer*), the distance between the singer and the hydrophone (*r*) and wind noise (*WN_psd_*) are the explanatory variables. *WN_psd_* is modelled as linear, *r* is modelled as nonlinear, *unit type* is modelled as a factor, and *singer* is included in the model as the random effect. *P*-values suggest that all variables tested are statistically significant.

covariate	estimate (dB *re* 1 µPa at 1 m)	s.e.	*t*	*p*
*β*_0_	147.29	5.63	26.14	<2 × 10^−16^
*unit type b2*	−3.68	1.00	−3.67	0.000273
*unit type c*	−5.56	0.63	−8.85	<2 × 10^−16^
*unit type e*	1.82	0.62	2.94	0.003441
*unit type f*	−2.12	0.66	−3.23	0.001344
*unit type g*	−0.95	0.75	−1.27	0.204159
*unit type h*	−5.56	0.91	−6.13	1.93 × 10^−9^
*unit type i*	−8.12	1.11	−7.29	1.41 × 10^−12^
*unit type l*	2.29	1.00	2.29	0.022582
*WN_psd_*	0.52	0.09	5.74	1.79 × 10^−8^

smoother	E. d.f.	R. d.f.	*F*	*p*
*s(r)*	2.872	2.872	93.46	< 2 × 10^−16^

**Figure 5.**
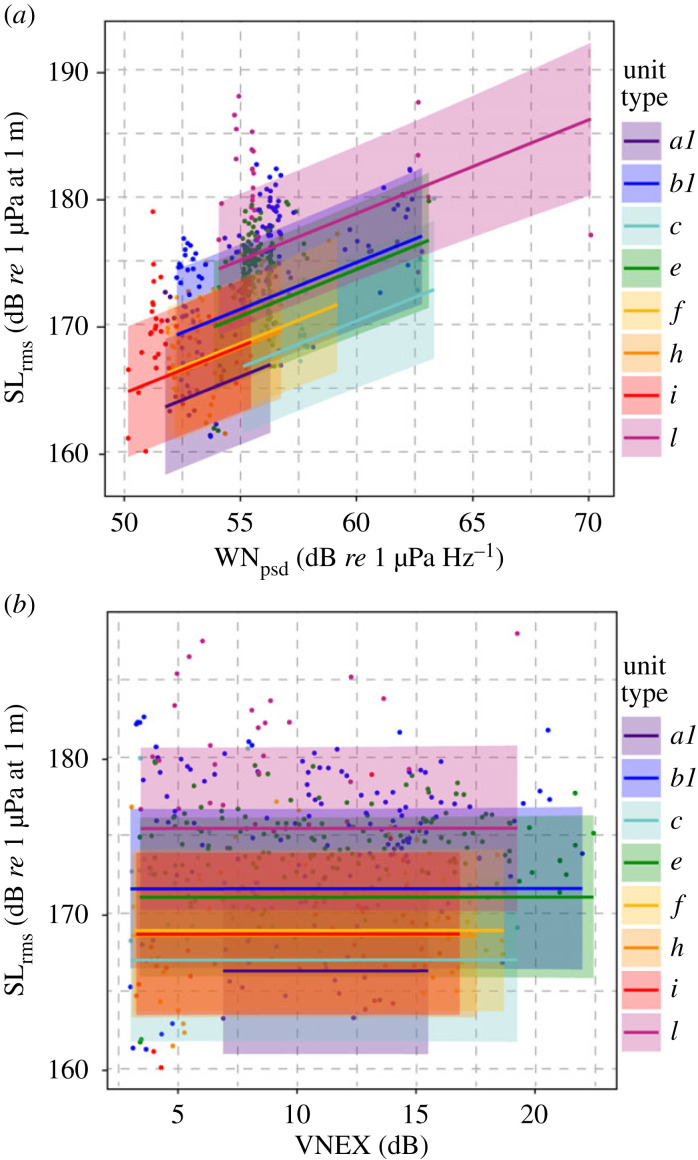
Plots showing the relationship between source levels of humpback whale songs and noise levels for the dataset containing both wind and vessel noise. Each unit type is represented by a different colour, with mean predicted values plotted as solid lines. The shaded areas represent the 95% pointwise confidence intervals. Observed values are shown as dots. The effect of the distance from the hydrophone, *r*, is not shown, which leads to the observed data appearing spread in this plot. Panel (*a*) shows that source levels of humpback whale songs (*SL_rms_*) are positively correlated to the underlying wind noise (*WN_psd_*). Panel (*b*) shows that *SL_rms_* are not correlated to additional noise generated by the vessel (*VNEX*).

For the vessel noise dataset, eight unit types were present with a large enough sample size (*N* ≥ 15) to be included in the analysis. Results of the statistical analyses for this dataset show that *WN_psd_* was still statistically significant (*p*-value = 1.71 × 10^−11^) and contributed to the model (AIC for the model without any noise = 2308.82; AIC for the model including *WN_psd_* = 2272.654). However, the vessel noise excess (*VNEX*) was not significant, whether it was modelled as linear (*p*-value = 0.97814) or nonlinear (*p*-value = 0.978) and did not contribute to the model (AIC for the model including both *WN_psd_* and *VNEX* modelled as linear = 2279.238; AIC for the model including both *WN_psd_* and *VNEX* modelled as nonlinear = 2276.107). Similarly to the previous dataset, the best model included only *unit type*, *singer*, the distance between the singer and the buoy (*r*), and *WN_psd_*. For this model, plots of residuals did not show any distinctive patterns. No overdispersion was found. To better visualize our findings, table 2 and figure 5 report the results for the model including *VNEX* as linear and show that, even in the presence of vessel noise, the singers are still adjusting the source levels of their songs in response to the underlying wind noise, and the vessel noise excess does not elicit an additional Lombard response.

**Table 2.** Parameter estimates and *p*-values for the model testing for a correlation between source level of humpback whale songs and noise levels for song units produced when both wind and vessel noise are present. In this model, source level of the units (*SL_rms_*) is the response variable; while unit type (*unit type*), singer ID (*singer*), the distance between the singer and the hydrophone (*r*), wind noise (*WN_psd_*) and vessel noise excess (*VNEX*) are the explanatory variables. *WN_psd_* and *VNEX* are modelled as linear, *r* is modelled as nonlinear, *unit type* is modelled as a factor, and *singer* is included in the model as the random effect. *P*-values suggest that *VNEX* is not statistically significant (*p*-value = 0.97814).

covariate	estimate (dB *re* 1 µPa at 1 m)	s.e.	*t*	*p*
*β*_0_	125.5	6.31	19.90	<2 × 10^−16^
*unit type* *b1*	5.29	0.86	6.12	2.09 × 10^−9^
*unit type c*	0.71	1.14	0.62	0.53646
*unit type e*	4.74	0.87	5.46	8.19 × 10^−8^
*unit type f*	2.60	0.97	2.68	0.00767
*unit type h*	2.28	1.13	2.02	0.04436
*unit type i*	2.38	0.98	2.43	0.01569
*unit type* *l*	9.14	1.03	8.90	<2 × 10^−16^
*WN_psd_*	0.74	0.11	6.91	1.71 × 10^−11^
*VNEX*	0.001	0.04	0.03	0.97814

smoother	E. d.f.	R. d.f.	*F*	*p*
*s(r)*	2.377	2.377	14.88	6.93 × 10^−7^

## 4. Discussion

Humpback whale singing behaviour evolved in an environment characterized by variable levels of noise generated by natural sources, and male humpback whales have been shown to adjust the source levels of their songs to compensate for an increase in wind noise levels [52,53]. However, human activities, such as shipping, can introduce additional noise in the ocean that can potentially interfere with humpback whale communication. This study investigated the response of humpback whale singers to increasing levels of vessel noise and compared it to their response to wind noise. Our results show that in the presence of vessel noise, singing humpback whales keep adjusting the source levels of their songs to compensate for variable levels of the undelaying wind noise; however, they do not show an additional Lombard response to vessel noise.

The main source of natural noise in our study area is the wind [53]. Wind noise was measured over the intercentile frequency range of each song unit. Considering the whole dataset, this spanned from 46 to 5094 Hz, encompassing the peak in the wind noise described by the Wenz curves which is between 100 Hz and 1 kHz [20,21]. Noise levels varied between 50 and 70 dB *re* 1 µPa Hz^−1^. This is consistent both with results of Girola *et al.* [53] that reported values of 53 to 71 dB *re* 1 µPa Hz^−1^, and with the Wenz curves for noise generated by winds between 0 and 37 km h^−1^ [20] which is the common wind range in our study area [78].

Our results show that as wind noise increases, the source levels of the song also increase which is consistent with results of Dunlop *et al*. [60] and Fournet *et al*. [54] for humpback whale social sounds, and with results of Girola *et al*. [53] and Guazzo *et al*. [52] for humpback whale songs. Our results also confirm a further finding from Girola *et al*. [53] showing that the increase is linear and an upper limit is not reached.

Our study also demonstrates that, although singing humpback whales adjust the source levels of their songs to compensate for variable levels of wind noise, they do not show a Lombard effect in response to vessel noise. Our results confirm findings by Dunlop [51], who showed no changes in the source levels of humpback whale social sounds in response to vessel noise, but are different from those of Fournet *et al.* [54], who reported a linear correlation between social sound levels and noise attributed to the passage of a large vessel. However, Fournet *et al*. [54] did not estimate received levels of vessel noise at the location of the whales. Instead, they assumed that noise recorded at the hydrophone during the passage of a ship a few kilometres away was dominated by vessel noise and was representative of the noise received by the vocalizing whales. Moreover, the effect of the underlying natural noise was not accounted for. Therefore, direct comparisons with our results are limited.

In our study, the absence of a Lombard response to vessel noise may suggest that humpback whales could be using other strategies to cope with this type of noise that are better suited to deal with its distinctive characteristics. While wind noise is diffused over large areas and is broadband, a vessel travelling in relative proximity of the singers can be considered a point source and the noise it produces is characterized by distinct tonal components. These differences may allow humpback whales to use different copying mechanisms, such as spatial release and/or comodulation release from masking.

Spatial release from masking is available to animals with binaural hearing that are capable of identifying the direction of a point sound source because the acoustic wave is received first on the ear closer to the source [79]. If signal and noise are spatially separated, a potential receiver can use directionality to discriminate between the two [80,81]. Several studies suggest that humpback whales can identify the location of calling conspecifics [65,67,82] and since vessel noise is in the same frequency range as humpback whale vocalizations, we can expect singers to be able to localize nearby vessels as well. Therefore, humpback whales may well have the hearing abilities required to use spatial release to overcome masking by vessel noise.

Comodulation masking release is a reduction in masking of a signal that occurs when the noise has an on-frequency component and it is also coherently amplitude modulated [83]. In this type of noise, the on-frequency component is centred on the frequency of the signal, while the off-frequency components, referred to as flanking bands, do not overlap with the signal, but they have amplitude fluctuations correlated to amplitude fluctuations of the on-frequency component [83]. In this case, masking is lower compared with when the noise is unmodulated or incoherently modulated. Vessel noise consists of continuous broadband noise generated by cavitation that is not comodulated, and tonal components produced by rotating and reciprocating machinery onboard the vessel, such as propellers blades, generators, engines, fans and pumps [25,26,84] that are comodulated [85]. Therefore, singing humpback whales may be able to use comodulation masking release to cope with vessel noise. Further research is needed to test these hypotheses.

To conclude, this project confirms results from previous studies showing that humpback whale singers increase the source level of their song to compensate for wind noise. The Lombard response to wind noise is maintained even when vessel noise is dominant, and vessel noise does not elicit an additional increase in source levels. Our results reinforce the importance of considering natural variability in animal behaviour and accounting for environmental factors when assessing the effect of an anthropogenic disturbance.

## Data Availability

The data are provided in electronic supplementary material [86].
